# Chemical Composition of Four Industrial Hemp (*Cannabis sativa* L.) Pollen and Bee Preference

**DOI:** 10.3390/insects14080668

**Published:** 2023-07-26

**Authors:** Beatrice N. Dingha, Louis E. Jackai

**Affiliations:** Department of Natural Resources and Environmental Design, North Carolina A&T State University, Greensboro, NC 27411, USA; lejackai@ncat.edu

**Keywords:** industrial hemp, *Cannabis sativa*, hemp, pollen, bees, pollinators, protein, amino acids, fatty acids, proximate analysis

## Abstract

**Simple Summary:**

Research on nutritional requirements in combination with improving bee protection has recently increased because of the continuous global decline in the abundance and diversity of bees. The decline is currently attributed to several stressors, including climate change, diminishing forage resources, and pesticide use, among other factors. Industrial hemp (*Cannabis sativa* L.) is a new crop that is grown over a wide geographic area in the United States, providing economic and nutritional benefits to humans. However, the contribution of hemp floral resources (pollen) to bee nutrition is not well understood. We investigated the chemical composition of pollen from four industrial hemp varieties (Canda, CFX-2, Henola, and Joey) and documented the abundance and diversity of bees on the crop using two sampling methods. Results showed differences in composition among the four hemp varieties. Overall, the Joey variety was the most preferred by bees, despite expressing lower protein, amino acid, and saturated and monosaturated fatty acid content. Based on our findings, we concluded that industrial hemp pollen provides some nutritional benefits to bees. However, it is important to understand that multiple sources of pollen are needed for sustained bee survival.

**Abstract:**

Apart from its economic value, industrial hemp (*Cannabis sativa* L.) is a prolific pollen producer, serving as a food source for bees. However, little is known regarding the extent to which varietal differences in hemp pollen chemistry influences bee preference. Here, we report the chemical profile of pollen from four hemp varieties (Canda, CFX-2, Henola, and Joey) and bee abundance and diversity, using direct visual counts and pan traps. The number and type of bees on each variety was recorded and the chemical composition (proximate analysis and mineral, amino, and fatty acid profiles) of pollen from each hemp variety was determined. During the entire sampling period, three bee types (bumble bees, honey bees, and sweat bees) were recorded, with a combined total of 1826. Among these, sweat bees and bumble bees were the most prevalent and were highest on the Joey variety. The four varieties expressed protein content ranging from 6.05% to 6.89% and the highest in Henola. Seventeen amino acids were expressed in all varieties, with leucine recording the highest content ranging from 4.00 mg/g in Canda to 4.54 mg/g in Henola. In general, Henola expressed high protein, amino acid, and saturated and monosaturated fatty acid contents and recorded significantly fewer bees compared with Joey, which had a low content of these components and a high content of polyunsaturated fatty acids. Our findings suggest that, while industrial hemp offers abundant and accessible pollen that would promote bee health and sustainability of their ecosystem services, the nutritional quality may not be adequate for bee growth and development as an exclusive pollen source.

## 1. Introduction

Industrial hemp *(Cannabis sativa* [L.]), which is native to central Northeast Asia, ranks amongst the oldest known cultivated crops in the world [1]. It is a multipurpose crop grown for flower, fiber, and seed, with several countries in Europe, Asia, and North and South America involved in the cultivation of the crop [2]. The inflorescences of the plant produce two major phytochemicals, including delta tetrahydrocannabinol (THC) (which has an intoxicating effect and cannabidiol (CBD) (which is non-intoxicating). Varieties with low tetrahydrocannabinol (THC) levels are grown primarily for the production of fiber and or grain [3]. The grains are used in the production of oils and foods, while fiber varieties are used for textiles, composite plastics, building materials, and paper [4]. Cannabidiol (CBD) has numerous applications in the pharmaceutical, cosmetic, and food industries [5]. Hemp production had been restricted because it can be mistaken for marijuana that usually contains higher levels (≤0.3%, the accepted threshold) of THC. The passing of the 2018 Farm Bill (which removed industrial hemp and its seeds from the Drug Enforcement Administration’s (DEA) schedule of controlled substances) resulted in the legalization of hemp production in the United States. Three years after its legalization, hemp production totaled USD 824 million, with an increase in fiber and grain acreage and yield [6].

Some hemp varieties have male and female flowers on the same plant (monoecious) or on separate plants (dioecious). Though both the male and female plants produce flowers, only the male plants have pollen sacs, which release copious amounts of pollen when mature. Industrial hemp is a wind-pollinated plant and varieties grown for CBD typically consist of all-female plants since pollination is undesirable as seed production reduces CBD yield. The flowers produce pollen but not nectar. Several different bee groups have been reported on industrial hemp flowers [7], suggesting that this may be a potential source of pollen for foraging bees. Pollinators, including bees, rely on floral resources (nectar and pollen) for reproduction, development, and survival [8,9,10]. While nectar is the main source of carbohydrates, pollen is the major source of proteins and lipids [8,10]. Pollen is considered to be more vital than nectar because it has an array of important nutrient components, including proteins, amino acids, lipids, vitamins, minerals, and trace elements, required by bees for their well-being [11,12,13,14]. Several studies have shown that the composition of amino acids and fatty acids varies among plant species [15,16,17,18,19,20]; pollen is reported to have 2.5% to 61% protein [21]. Such variations could influence plant–pollinator interactions that involve pollinators that pollinate plants while they forage on flowers for resources and rewards.

The decline in insect pollinators has been widely documented. This is of the greatest concern among honey bees and bumble bees, which pollinate about USD 15 billion worth of crops in the United States annually [22,23,24,25,26,27]. The decline has been attributed to multiple stressors, including the degradation and fragmentation of natural habitats, climate change, loss of flower-rich plant communities, the spread of pathogens and parasites, and inappropriate use of agricultural pesticides [28,29,30,31]. However, degradation and fragmentation of habitats resulting in the loss of floral resources is considered a major cause of pollinator decline [32]. Current and ongoing mitigation efforts involve actions targeting management strategies to support and sustain pollinator abundance and diversity [33,34,35]. Industrial hemp is a crop that has become increasingly lucrative and is gaining popularity worldwide. In the United States, its cultivation has increased in acreage since its legalization. For instance, hemp acreage increased from zero in 2013 to over 90,000 acres in 2018, the highest acreage since 1943 when about 146,200 acres of hemp were planted [36]. In 2021, the area harvested for grain and fiber was estimated at over 24,000 acres [6]. In addition, growers are turning towards scaling up the cultivation of fiber and grain varieties [6]. This prompted us to embark on the current study to investigate the chemical composition of industrial hemp pollen and to document the diversity and abundance of bees on the crop.

## 2. Materials and Methods

### 2.1. Experimental Design

A field experiment was conducted in 2021 at the North Carolina Agricultural and Technical State University (NC A&T SU) Research Farm in Greensboro, North Carolina, United States (36.0638452, −79.7438869). We used four dual-purpose industrial hemp varieties (Canda, CFX-2, Henola, and Joey), grown for grain and fiber and among the most popular varieties that are grown in the United States. Seeds were purchased from King’s AgiSeeds Inc. (1828 Freedom Road, Lancaster, PA, USA). The experiment was set up in a randomized complete block design (RCBD) with four replications. Each experimental unit consisted of 10 rows, each 7 m long with 7.5 inches between rows. Seeds were planted on 18 May 2021 using a planter (EarthWay^®^ Precision Garden Seeder, Model 1001B) at a depth of approximately 0.01–0.015 m below the soil surface, spaced at 0.4 m apart within rows, 0.19 m between rows, and 2.0 m between treatments. General agronomic practices such as weed control and irrigation were carried out *ad libitum* and no insecticide was applied.

### 2.2. Insect Sampling

**Figure 1:**
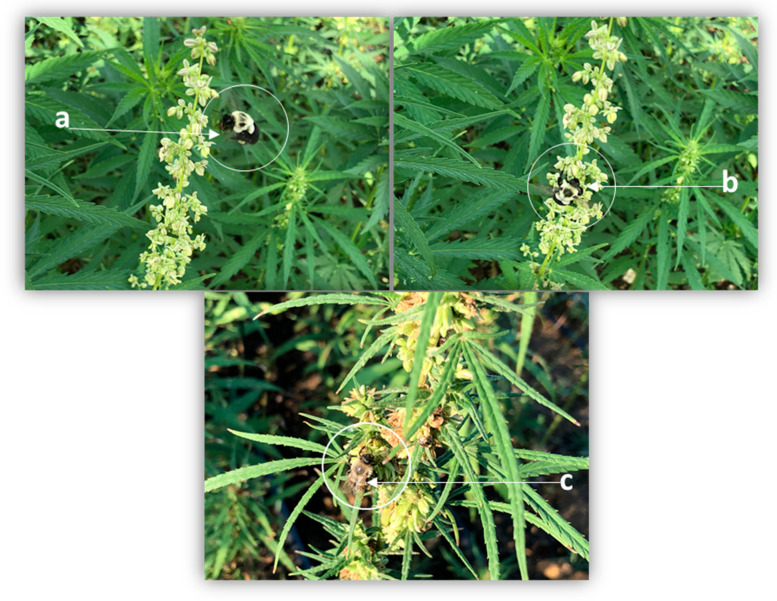
Bumble bee landing on industrial hemp flower (**a**) bumble bee (**b**) and sweat bee (**c**) foraging on industrial hemp flower.

We sampled for specific pollinators (honey bees, bumble bees, and sweat bees) that were observed and documented on industrial hemp (Figure 1), using direct visual counts and pan traps.

#### 2.2.1. Direct Visual Counts

This was carried out as conducted by [37,38], where the number of pollinator visitors on the ten rows of each industrial hemp variety was identified and counted using “snapshot”, in which the number of insects were counted visually for 60 s. These counts were conducted weekly for pollinators between 9:00 and 11:00 a.m., for seven weeks starting 21 June when the plants began to flower and repeated weekly for seven weeks.

#### 2.2.2. Assessment of Pollinators Using Pan Traps

Sampling of pollinators was conducted using pan traps of three different colors (blue, white, and yellow), as described by [37,38]. Briefly, the traps were made up of 16 oz squat polypropylene deli bowls painted with UV-bright fluorescent blue (blue trap) or yellow paint (yellow trap) and unpainted 12 oz white Styrofoam bowls (white trap). Trap setup comprised gluing individual unpainted 16 oz polypropylene deli bowls onto a 36” metal prop. Three of these were placed 0.2 m apart between the 10 rows of each treatment during the entire sampling period. Sampling began on 24 June 2021, at which time each colored bowl (blue, yellow, and white) was then placed inside one of the unpainted glued bowls on a prop and filled with a soapy water solution. Traps were placed between 08:00 and 10:00 a.m. and collected after 24 h following the order they were placed to ensure that all traps were available to insects for roughly the same duration. This was repeated weekly for seven weeks. Each pan trap was drained and the contents placed in a vial containing 70% ethanol. Samples were transported to the laboratory and stored in a refrigerator for later identification.

### 2.3. Pollen Sample Collection and Analysis

#### 2.3.1. Pollen Collection

**Figure 2:**
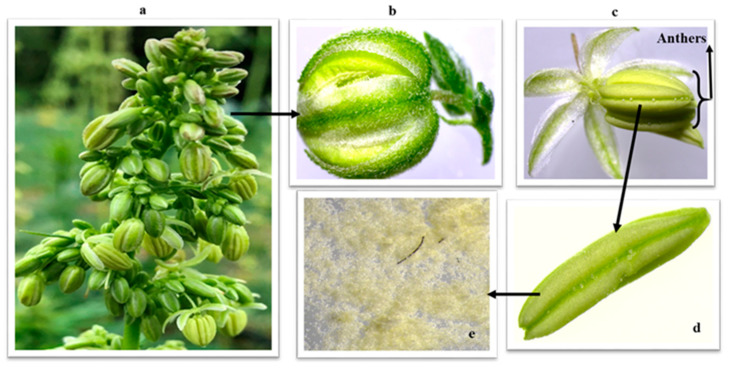
Flowers of industrial hemp showing (**a**) cluster of flowers at magnification 1.25×; (**b**) close-up of one immature flower; (**c**) mature flower showing pollen sacs and some pollen grains on the outside of the anthers at magnification 1.25×; (**d**) pollen sac containing pollen grains; (**e**) pollen grains at 4× magnification.

The small flower and pollen size made the direct collection of pollen from inflorescences difficult. Therefore, at flowering, approximately 500 g of mature hemp flowers (Figure 2) of each variety were harvested and placed in small brown paper bags that were then put in Ziplock^®^ bags and placed in Styrofoam shipping containers with dry ice. Samples were shipped to a commercial analytical laboratory (Eurofins Food Chemistry Testing 6304 Ronald Reagan Ave., Madison, WI, USA) for analysis of moisture, crude fiber, protein, ash, mineral content, amino acids, and fatty acids.

#### 2.3.2. Chemical Analysis

1 Proximate chemical composition

Proximate analysis to assess moisture, ash, crude fiber, and protein was performed on pollen samples shipped to the Eurofins Food Chemistry Testing laboratory. The analysis was carried out according to the Association of Official Analytical Chemists (AOAC)’s methods as follows: The moisture content was ascertained using AOAC methods 925.09 and 926.08 [39] by drying each sample in a vacuum oven at 100 ± 5 °C for 5 h. Ash content was determined using AOAC method 923.03 [40] by incinerating pollen samples in an electric furnace at 550 °C. AOAC method 968.06 [41] was used to calculate the percentage of crude protein by multiplying the percentage of nitrogen content obtained by a factor of 6.25. Crude fiber was quantified using AOAC method 962.09 [42] gravimetrically from a 1 g sample.

2 Mineral content

Mineral elements boron (B), calcium (Ca), potassium (K), sodium (Na), magnesium (Mg), iron (Fe), zinc (Zn), and copper (Cu) concentrations were determined using ash obtained from ash content determination following AOAC methods 984.27, 985.01, and 2011.14 [43] by inductively coupled plasma optical emission spectroscopy (ICP-OES).

3 Amino acid composition

Seventeen amino acids, including aspartic acid, threonine, serine, glutamic acid, proline, glycine, alanine, valine, isoleucine, leucine, tyrosine, phenylalanine, lysine, histidine, arginine, cysteine, and methionine, were analyzed from the four industrial hemp varieties. The Eurofins Food Chemistry Testing Laboratory reference method used for this analysis was amino acids TAALC_S, as described by Henderson and Brooks [44].

4 Fatty acid composition

The fatty acid composition as saturated and unsaturated fatty acids (cis- and trans- isomers) and as fatty acid methyl esters (FAMEs) was measured by Eurofins Food Chemistry Testing laboratory according to the official methods and recommended practices of the AOC method 996.06 [45], AOAC methods Ce 2b-11 (alkali hydrolysis), Ce1j-07 (gas chromatography with flame ionization detection (GC-FID)), and Ce 2-66 (for methyl esters of fatty acids) [46]. After adding a known amount of internal standard, fatty acids were saponified and methylated and methyl esters were extracted. Then, the methyl esters of the fatty acids were analyzed by capillary gas chromatography using external standards for quantification. From the measured fatty acid composition, the following classes of fatty acids were summed: saturated fatty acids, monounsaturated fatty acids, polyunsaturated fatty acids, fatty acids, and total trans and cis fatty acids.

### 2.4. Data Analysis

All analyses were conducted using JMP Pro (JMP Pro v. 14 SAS Institute, Cary, NC, USA). For both sampling methods, the total number of insects belonging to each pollinator type (honey bees, bumble bees, and sweat bees) captured throughout the sampling period was obtained. Plant height data from 80 randomly selected plants at flowering were recorded. Analysis of variance (ANOVA) was used to examine differences in plant height and the distribution of pollinators among the four industrial hemp varieties. Means were separated using Tukey–Kramer honestly significant difference at *p* = 0.05. Regression analysis was used to describe the relationships between plant height and pollinator types and total number of pollinators. Principal component analysis (PCA) was used to identify common factors that accounted for most of the variations in the chemical composition data (crude fiber, protein, ash moisture, protein, amino acid, minerals, and fatty acids) and to also examine relationships between the different independent variables of each chemical component. It also allowed us to determine the eigenvectors that maximized the variance. Thereafter, it attained a second linear function PC2 that was uncorrelated with PC1. The PCA data were not standardized since the values were similar, indicating similar variance.

## 3. Results

Three pollinator types (bumble bee, honey bee, and sweat bee) were recorded from both sampling methods on all four industrial hemp varieties during the sampling period. In total, 1826 pollinators were recorded, of which 84.7% were sweat bees, 14.6% bumble bees, and 0.8% honey bees.

**Figure 3:**
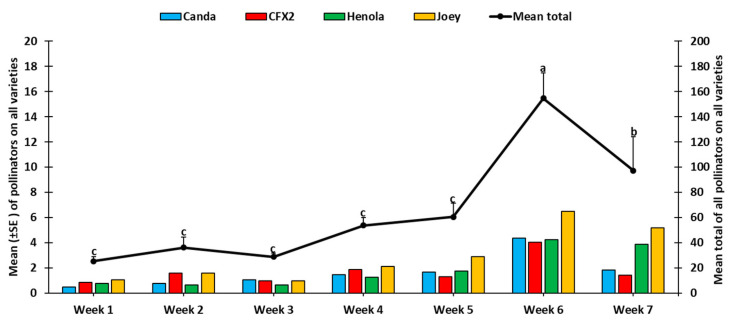
Weekly distribution of pollinators recorded from both visual and pan traps among the four industrial hemp varieties. Mean numbers with the same letters are not significantly different (*p* > 0.05).

Combining the pollinator catches for each sampling week showed a highly significant difference (F_6, 890_ = 29.4, *p* < 0.0001) among the sampling weeks, with a steady increase from the first sampling week (25.25 ± 3.79) through the fifth week, with the highest peak occurring on week six (154.75 ± 19.90) (Figure 3). During the first six weeks, the catch among the four hemp varieties was not significantly different; however, during the seventh week, the number of pollinators among the four varieties was significantly different (*F*_3, 123_ = 5.36, *p* < 0.0017); Joey recorded the highest number and CFX-2 the lowest (Figure 3).

**Figure 4:**
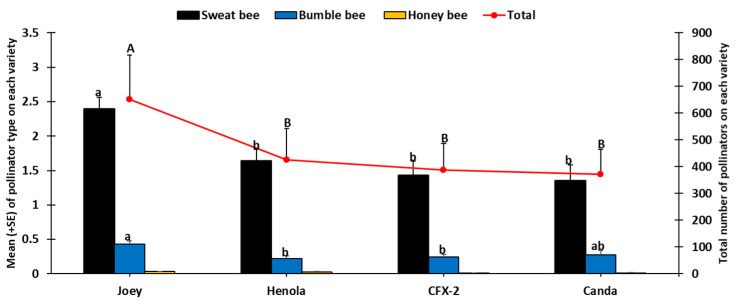
Abundance of pollinator types (bars) and total pollinators (line) from both visual and pan traps among the four industrial hemp varieties during the entire sampling period. Lowercase letters are used to compare means of each pollinator type among the four industrial hemp varieties. Uppercase letters are used to compare combined pollinator types among the four varieties. Means followed by the same letters are not significantly different (*p* > 0.05).

Our results show that there was a significant difference (*F*_3, 908_ = 5.10, *p* < 0.0005) in the number of sweat bees among the four industrial hemp varieties, with Joey recording the highest and Canda the lowest number (Figure 4). Similarly, the number of bumble bees was significantly different among the four hemp varieties (*F*_3, 908_ = 3.86, *p* < 0.0092), with Joey recording the highest number (Figure 4). However, the number of honey bees was not significantly different (*p* = 0.05) among the four industrial hemp varieties (Figure 4). Combining all three pollinator types, there were significant differences (*F*_3, 908_ *=* 7.06, *p* < 0.0001) among the four industrial hemp varieties. The highest number of pollinators was recorded on Joey (651 ± 166.58) and the lowest on Canda (372 ± 93.72) (Figure 4).

Plant height at flowering ranged from 0.25 to 1.12 m in CFX-2, 0.38 to 1.40 m in Joey, 0.41 to 1.21 m in Canda, and 0.36 to 0.94 m in Henola. On average, plant height was significantly different (F3, 316 = 29.4, *p* < 0.0001) among the four varieties, with Joey being the tallest variety (0.76 ± 0.3 m) > Canda (0.68 ± 0.02) > Henola (0.56 ± 0.01) and CFX-2 (0.52 ± 0.02) the shortest. Regression analysis indicated that there was a non-significant relationship between each pollinator type and plant height (for bumble bees (y = −0.19 + 0.76x; *n* = 4; r^2^ =0.76; *p* = 0.13), honey bees (y = −0.02 + 0.06x; *n* = 4; r^2^ =0.21; *p* = 0.54), and sweat bees (y = −0.10 + 2.85x; *n* = 4; r^2^ =0.42; *p* = 0.35)) and between total pollinator count and plant height (y = −0.33 + 3.73x; *n* = 4; r^2^ =0.49; *p* = 0.30).

As shown in Table 1, the percentage of moisture, ash, crude fiber, and protein content of pollen varied among the four industrial hemp varieties. Moisture content ranged from 76.8% to 81.6%, with Henola recording the least (Table 1). The crude fiber content ranged from 1.5% to 2.67%, with the highest (2.67%) recorded in Henola. Similarly, protein content ranged from 6.05% to 6.89%, with the highest in Henola (Table 1) compared with the other varieties. Conversely, ash content ranged from 1.55% to 1.87% and was higher in CFX-2 than in the other varieties.

A total of 17 amino acids were detected in the pollen of all four industrial hemp varieties and, of these, 9 were essential amino acids (isoleucine, leucine, valine, arginine, lysine, phenylalanine, threonine, histidine, and methionine) and 8 were non-essential amino acids (alanine, aspartic acid, cystine, glutamic acid, glycine, proline, serine, and tyrosine) (Table 2). Analysis of the amino acid composition revealed that, for each hemp variety, leucine content was the highest, with leucine > valine > isoleucine; however, these three essential amino acids were highest in Henola relative to the other varieties (Table 2).

Also, as shown in Table 2, lysine content was highest among the intermediate essential amino acids for each hemp variety. However, Henola recorded the highest content (3.96 mg/g) among the four varieties. The intermediate essential amino acids included lysine > arginine > threonine > phenylalanine for Henola. Meanwhile for Canda, CFX-2, and Joey, lysine > arginine > phenylalanine > threonine (Table 2). Among the non-essential amino acids, aspartic acid was the highest in each variety > glutamic acid > proline > alanine > serine > glycine > tyrosine > cysteine (Table 2).

Our results revealed that fatty acids in the four general groups (saturated, monounsaturated, polyunsaturated, and trans fats) were detected in the pollen of all four hemp varieties (Table 3). Among the saturated fatty acids, palmitic acid (16:0), steric acid (18:0), arachidic acid (20:0), behenic acid (22:0), and lignoceric acid (24:0) were the main fatty acids detected and ranged from 0.125% to 0.135%, 0.14% to 0.022%, 0.008% to 0.016%, 0.009% to 0.019%, and 0.008% to 0.013%, respectively (Table 3). In addition, all four hemp varieties expressed low content (<0.007%) of the following saturated fatty acids 4:0 butyric acid, 6:0 caproic acid, 8:0 caprylic acid, 10:0 capric acid, 12:0 lauric acid, 14:0 myristic acid, 15:0 pentadecanoic, 15:1 pentadecanoic, 17:0 heptadecanoic acid, and 17:1 heptadecenoic acid. Among the varieties, the composition varied and 16:0 palmitic acid, 18:0 steric acid, and 24:0 lignoceric acid were more prominent in pollen from Henola > CFX-2 > Canda = Joey (Table 3).

All four hemp varieties had low content (<0.007%) of the following monounsaturated fatty acids: 14:1 myristoleic acid, 16:1 palmitoleic, 20:1 eicosenoic, 22:1 erucic acid, and 24:1 nervonic acid; however, 9c 18:1 oleic acid was the only monounsaturated fatty acid >0.0007% in all four varieties and was highest in Henola (0.034%) (Table 3). The following polyunsaturated fatty acids, including 18:3 gamma linolenic acid, 18:3 alpha linolenic acid, 18:4 octadecatetraenoic, 20:2 eicosadienoic acid, 20:3 eicosatrienoic acid, 20:3 homo-gamma-linolenic acid, 20:4 arachidonic acid (n3), 20:5 eicosapentaenoic acid, 21:5 heneicosapentaenoic acid, 22:2 docosadienoic acid, 22:3 docosatetraenoic acid, 22:4 docosatetraenoic, 22:5 docosapentaenoic (n3), 22:5 docosapentaenoic (n6), and 22:6 docosahexaenoic, were detected in low content (<0.007%) in all the varieties. However, the polyunsaturated fatty acids, 18:2 linolenic acid, 18:3 alpha linolenic acid, omega 3 fatty acid, omega 6 fatty acid, and omega 9 fatty acid were expressed in all the hemp varieties (Table 3). Omega 3 and omega 6 fatty acids were higher in Joey (0.141% and 0.171%, respectively) compared with the other varieties, while Omega 9 fatty acid was higher in Henola relative to the other hemp varieties (Table 3).

All four hemp varieties expressed four types of fatty acids (saturated, monounsaturated, polyunsaturated, and trans fatty acids), with low content (<0.007%) detected in trans fatty acids in all four hemp varieties. Overall, saturated, monounsaturated, and polyunsaturated fatty acids were relatively higher in Henola than the other industrial hemp varieties (Table 3). All four hemp varieties expressed low content (<0.007%) of the following: total 18:1, total 18:2, and total 18:3 trans; however, total 18:1 cis and total fatty acids were relatively higher in Henola compared with the other varieties, while total cis unsaturated fatty acid was higher in Joey compared with the other varieties (Table 3).

The following ten macro and micro minerals (boron (B), calcium (Ca), copper (Cu), iron (Fe), magnesium (Mg), manganese (Mn), phosphorus (P), potassium (K), sodium (Na), and zinc (Zn)) were analyzed from the four hemp varieties (Table 4). Among them, potassium ranged from 5.4 mg (Joey) to 5.8 mg (CFX-2); calcium ranged from 1.06 mg (CFX-2) to 1.25 mg (Canda); manganese ranged from 0.711 mg (Joey) to 0.955 mg (Henola). Sodium was < 0.025 in all four hemp varieties (Table 4).

The principal component analysis (PCA) indicated that the first two principal components accounted for about 98% of the variability (Table 5). The PC1 was interpreted primarily as an index of crude fiber that ranged from 1.50 to 2.67% and PC2 was primarily an index of ash ranging from 1.55 to 1.87%. For amino acid, the PCA showed that the first two PCs would explain approximately 95.4% of variability (Table 5).

PC1 was interpreted as an indicator of arginine and lysine and PC2 of valine (Table 6). Table 5 shows that the first two principal components of fatty acid explained about 99% of the variation. In this case, PC1 was an index of monounsaturated fatty acids and total 18:1 cis and PC2 was an index of 18:2 linoleic acid and omega 6 fatty acids. The first two PCs for mineral accounted for 87.4% of the variation and eigenvalues for all three PC scores were greater than 1.0 (Johnson, 1998). We interpreted PC1 as a combination of iron, magnesium, and manganese and PC2 as copper (Table 6).

**Figure 5:**
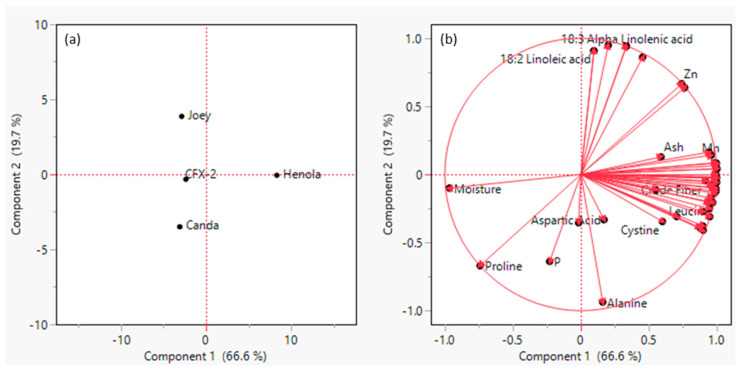
Principal component analysis (PCA) of the chemical composition of pollen of four industrial hemp varieties showing (**a**) score and (**b**) plot.

The resulting principal component analysis (PCA) score plot (Figure 5a) and loading plot (Figure 5b) display the pattern of covariation among the four industrial hemp varieties and the variables analyzed. Our results identified two principal components 1 and 2 that explained 86.6% of the total variance. The first principal component (PC1) explained 67.3% of the data variability and allowed the separation of Henola, which was associated with a higher content of most of fatty acid components, including saturated fatty acids, iron, crude fiber, 18:0 steric acid, and 24:0 lignoceric acid. CFX-2, on the other hand, appeared to be related to higher moisture and potassium content. The second principal component, representing 19.3% of the data variability, showed the separation of Joey associated with high omega 6 fatty acid and Canda associated with higher proline and phosphorous content.

## 4. Discussion

Industrial hemp cultivation is rapidly expanding in the United States as a promising economically important crop for farmers, as well as a plentiful pollen source for bees and other pollinators. In this study, we documented the number and diversity of bees on four industrial hemp varieties. We also determined the chemical content of pollen from the four varieties. In total, we counted 1826 pollinators during the study, representing three types of bees, namely honey bees, bumble bees, and sweat bees. The community of bees documented here was similar to that of previous work with the same bee types (honey bees, bumble bees, and sweat bees) [47,48]. However, another study [7] indicated additional bee species, including the longhorn bee, miner bee, and leafcutter bee. Sweat bees were the most abundant bees in our study, accounting for 84.7% of the bee community on all four hemp varieties (Figure 4). In other studies, the honey bee was the most abundant, comprising nearly 60% [7] and 30% [48] of the bee community on industrial hemp. Several factors could have played a role in influencing the abundance and type of bee recorded. For example, the experimental field for this study was located at a university research farm in a county where agriculture is not intensive, with no beehives around the farm. Furthermore, the experimental plots were about two miles from all other experiments within the 492-acre research farm to prevent cross contamination of hemp research focused on cannabidiol (CBD). However, the experimental field in the study by [7] was located near active honey bee colonies. Additionally, both studies by [7,48] were conducted in regions where agriculture is intensive and may have had fields with sunflower and cucurbits in the nearby vicinity. These plants shed large amounts of pollen and also attract bees and other pollinators. The sampling method used could also have contributed to this variation. The commonly used standardized pan traps have been shown to be effective in attracting sweat bees [37,49,50,51]. However, their effectiveness varies with trap color, which may have led to skewed catch assessment. For example, the highest number of sweat bees were captured in white traps [37,50], blue traps [52], and yellow traps [53]. Our results are based on a combination of three colored pan traps (blue, white, and yellow), as well as direct visual counts, whereas [7] used only the blue pan trap and [48] used sweep nets. Despite the differences in captured bee species, our findings indicating high numbers of sweat bees in hemp plots were significant, given that attention is now beginning to focus on wild bees. Wild bees improve pollination efficiency twice as much as honey bees in several important crops, resulting in increased fruit sets [54]. Evidence of the complementary effect of wild bees and managed bees for enhanced crop pollination [55,56] signifies their importance in mitigating the effects of widespread decline in managed bee populations. The observed increase in the number of foraging bees recorded on industrial hemp across the sampling period (Figure 3) could be an indication of their quest to explore the pollen resources of industrial hemp as flowers of other late-season crop senesce in late summer.

Pollen is still the major source of proteins, amino acids, vitamins, lipids, and minerals, while nectar provides the main source of carbohydrates for bees. The content of proteins, amino acids, carbohydrates, and lipids (nutritional value) varies across plant species [15] and for protein, this variation ranges from about 2.0 to 60% [57]. The four industrial hemp varieties in our study had protein content within this range from 6.05% (Joey) to 6.89% (Henola) (Table 1). The challenge for foragers is the ability to search for the appropriate nutrients from diverse floral plant species. Some studies suggest that bees do not randomly collect pollen; some species, such as bumble bees [58,59,60,61,62,63] and honey bees [64], forage preferentially on pollen with high protein content. This did not appear to be the case in our study, as shown by the fact that the highest number of bumble bees were recorded on Joey, the variety with the lowest protein content. Our results are in agreement with those reported by other authors who have evaluated preference both in the field and in the laboratory [65,66,67]. It is also possible that, during foraging, bees would be selective and not simply collect pollen from all available plant species, with foragers showing a preference for some pollen types over others [18,68,69]. Other compounds may also have a role in this.

In addition to protein, pollen contains about 1.0% to 6.5% ash and diverse minerals [70,71]. All four industrial hemp varieties contained boron, calcium, copper, iron, magnesium, manganese, phosphorus, potassium, sodium, and zinc in varying concentrations. Among them, potassium was the most predominant element ranging from 5.4 mg (Joey) to 5.8 mg (CFX-2) (Table 4). Similarly, [71,72,73,74] found potassium to be the most dominant mineral in bee-collected pollen and pollen of other plants, such as some eucalyptus species. Ash content is a significant component in the honey bee diet; it was lowest in Canda (1.55%) and a little less than 2% in Henola (1.86%) and CFX-2 (1.87%). According to [75], adding 1.0% pollen ash to a synthetic diet remarkably increased brood rearing. The authors recommended a diet containing 1000 ppm potassium, 500 ppm calcium, 300 ppm magnesium, and 50 ppm each of sodium, zinc, manganese, iron, and copper because concentrations that were too low or too high could affect brood rearing. Although it has not been widely researched, potassium, calcium, and magnesium could be the most important minerals in the honey bee diet.

Furthermore, pollen generally contains all the essential amino acids (EAAs) [57] that bees must obtain from their diet. Isoleucine, leucine, valine, arginine, lysine, phenylalanine, threonine, histidine, methionine, and tryptophan are essential amino acids, whereas tyrosine, cysteine, serine, hydroxyproline, alanine, glycine, and proline are non-essential amino acids [76]. According to [76], the proportion (g per 16g N) of amino acid requirement for bees include threonine (3%), valine (4%), methionine (1.5%), leucine (4.5%), isoleucine (4%), phenylalanine (2.5%), lysine (3%), histidine (1.5%), arginine (3%), and tryptophan (1%). Among the EEAs, leucine, isoleucine, and valine are required by bees in the following quantity: leucine > isoleucine > valine [76]. From our study, seventeen amino acids, including nine EEAs, were reported in all four hemp varieties in varying amounts (Table 2). However, the quantity and quality of EAAs may not be critical factors influencing bee preference. Foraging honey bees were reported to show preference for rape pollen that is rich in leucine, isoleucine, and valine compared with field bean pollen [68], even though the content (≤0.4 mg/g) in both plants was low compared with the content and the intra-specific differences reported in our findings from hemp. In addition, some authors have reported that sunflower pollen is of low quality with low amounts of some amino acids, including methionine, glutamic acid, and proline, yet the flowers are highly attractive to bees [57,66,77,78,79,80]. Some works have suggested that some macronutrients [18,81,82,83], the ratio of lipids and proteins [18], and high lipid levels [84] may be the key drivers of bee foraging preference. This shows there is no scientific unanimity yet regarding the principal drivers of bee preferences.

We reported fatty acids in four general categories (saturated, monounsaturated, polyunsaturated, and trans fats) in pollen from all four hemp varieties (Table 3). Generally, the fatty acid content in pollen ranges from 1% and 20% depending on plant species [85]. In our study, the total fatty acids varied among the four hemp varieties and ranged rather low in comparison, from 0.438% in Joey to 0.537% in Henola (Table 3). Linoleic and linolenic acids (polyunsaturated fatty acids), oleic (monosaturated fatty acid), and palmitic (saturated fatty acids) are all components reported to satisfy the nutritional needs of bees and other pollinators [86,87,88,89,90,91]. Linoleic and linolenic acids were higher in Joey, the variety that recorded the highest number of bees while palmitic and oleic acids were higher in Henola which had the second highest. Pollen from different plant species may differ in composition and content but have some nutritional value to contribute toward the growth, development, and survival of bees. This may explain why some pollinators have a wide foraging range. For example, pollen dominated by linoleic and linolenic acids may play a significant role in inhibiting the growth of spore-forming bacteria and microbes that inhabit the brood combs of beehives [85]. Sunflower pollen, even though it is of low quality, recorded higher linoleic acid content [84,86,92], increased the longevity of young worker bees [79], and reduced *Crithidia bombi* infection in bumble bees and *Nosema ceranae* in honey bees [93] compared with rape pollen. Clearly, there is a lot about bees and pollinator foraging that is not fully understood.

While clues of the effects of pollen composition on bee foraging preference abound in the literature, results are inconsistent. Several factors may be responsible for this, including the source and form or type of the pollen. For example, in some studies, bee foraging preference was assessed using bee pollen or pollen from bee corbiculate (pollen baskets) that has been transformed due to the addition of nectar [9,15]. In others, foraging preference was correlated to the levels of particular nutrients in fresh pollen from the different plant species around the bee foraging landscape [55,60,62,63]. Yet, in others, the pollen source was enriched with protein [67] or modified protein [66]. Apart from these differences, pollen characteristics other than the nutritional quality appear to contribute to the observed preferences. For example, preference based on the pH of the pollen [94], phago-stimulants [95], phago-deterrents [96], and pollen toxins [97] may also influence bee foraging behavior. In addition, flower traits such as floral scent could also be used by bees to locate pollen sources and discriminate between different flower species [98]. Industrial hemp produces a strong floral scent due to the presence of volatile organic compounds (VOCs), which play a vital role in plant–insect communication [99]. Of such volatile organic compounds, thirty-five were identified from six hemp varieties and no variety emitted all the compounds; however, β-Myrcene was the most abundant compound [100] and is thought to be relevant in pollinator attraction [101], specifically for bumble bees [102]. Since industrial hemp produces copious amounts of pollen, the preference could also be related to the quantity and not the quality of the pollen.

## 5. Conclusions

Bees are essential to the functioning of ecosystems by enabling the reproduction of many plant species. In return, they benefit by collecting nutrients (nectar and pollen) required for their growth and health. Unfortunately, bee populations are declining because of the combined effects of climate change, loss of floral abundance and diversity, intensive agriculture, pests and pathogens, and pesticide usage. Industrial hemp is an emerging source of pollen for bees. Our findings indicate that the nutritional composition and content of industrial hemp pollen vary among hemp varieties and may not have been the major factor driving bee attractiveness to the crop. Nevertheless, pollen from these hemp varieties contains important nutrients that can contribute and add significant value to the food resources of diverse populations of bees for sustained survival.

## Figures and Tables

**Table 1:** Proximate analysis of pollen from four varieties of industrial hemp.

	Proximate Content (%)			
	Moisture	Ash	Crude Fiber	Protein
Canda	81.6	1.55	1.50	6.41
CFX-2	80.0	1.87	2.03	6.10
Henola	76.8	1.86	2.67	6.89
Joey	80.9	1.63	1.50	6.05

**Table 2:** Amino acid composition (mg/g) of pollen from four industrial hemp varieties.

			(mg/g)			
Amino Acids			Canda	CFX-2	Henola	Joey
**Essential amino acids**	Most essential	Isoleucine	2.46	2.39	2.73	2.33
		Leucine	4.00	3.87	4.54	3.84
		Valine	3.06	2.94	3.29	2.88
	Intermediate essential	Arginine	2.90	2.76	3.27	2.80
		Lysine	3.53	3.31	3.96	3.38
		Phenylalanine	2.34	2.33	2.69	2.29
		Threonine	2.32	2.30	3.59	2.25
	Least essential	Histidine	1.15	1.09	1.22	1.09
		Methionine	1.08	1.06	1.19	1.06
**Non-essential** **amino acids**	Non-essential	Alanine	3.29	3.10	3.18	3.02
		Aspartic Acid	8.32	7.09	7.97	7.81
		Cystine	0.658	0.607	0.667	0.632
		Glutamic Acid	5.54	5.35	6.55	5.74
		Glycine	2.50	2.41	2.72	2.41
		Proline	3.87	3.79	3.65	3.71
		Serine	2.72	2.64	3.01	2.68
		Tyrosine	1.78	1.69	2.04	1.75

**Table 3:** Fatty acid (%) profile of pollen from four industrial hemp varieties.

		(%)			
	Fatty Acids	Canda	CFX-2	Henola	Joey
Saturated	16:0 Palmitic acid	0.125	0.127	0.135	0.125
	18:0 Steric acid	0.014	0.015	0.022	0.014
	20:0 Arachidic acid	0.008	0.010	0.016	0.009
	22:0 Behenic acid	0.009	0.011	0.019	0.010
	24:0 Lignoceric acid	0.008	0.009	0.013	0.008
Mono-saturated	9c 18:1 Oleic acid	0.019	0.020	0.034	0.020
Polyunsaturated	18:2 Linoleic acid	0.137	0.138	0.152	0.171
	18:3 Alpha Linolenic acid	0.109	0.122	0.133	0.141
	Omega 3	0.109	0.122	0.133	0.141
	Omega 6	0.137	0.138	0.152	0.171
	Omega 9	0.019	0.020	0.034	0.020
Fatty acid types	Saturated	0.157	0.164	0.195	0.158
	Monounsaturated	0.027	0.028	0.045	0.029
	Polyunsaturated	0.236	0.249	0.272	0.298
Total	Total 18:1 cis	0.028	0.029	0.047	0.030
	Total Cis Unsaturated Fatty Acids	0.263	0.277	0.317	0.327
	Total Fatty Acids	0.438	0.461	0.537	0.508

**Table 4:** Mineral composition (mg) of pollen from four industrial hemp varieties.

Variety	Minerals (mg)								
	B	Ca	Cu	Fe	Mg	Mn	P	K	Zn
Canda	0.006	2.04	0.003	0.016	0.770	0.006	1.25	5.53	0.012
CFX-2	0.008	2.83	0.002	0.019	0.832	0.009	1.06	5.85	0.012
Henola	0.008	2.74	0.006	0.003	0.955	0.013	1.11	5.57	0.013
Joey	0.006	2.00	0.002	0.002	0.711	0.007	1.11	5.40	0.013

**Table 5:** Eigenvalues, proportion of variance and cumulative variance explained by principal component analyses (PCA) for independent variables measured from four industrial hemp varieties.

			Variance	
Component	PC	Eigenvalue	Percent	Cumulative Percent
Proximate analysis	1	3.23	80.9	80.9
	2	0.72	17.9	98.8
	3	0.05	1.2	100.0
Amino acids	1	13.9	81.9	81.9
	2	2.3	13.5	95.4
	3	0.79	4.60	100.0
Fatty acids	1	11.70	68.8	68.8
	2	5.13	30.2	99.0
	3	0.17	1.0	100.0
Minerals	1	6.03	67.0	67.0
	2	1.84	20.4	87.4
	3	1.13	12.6	100.0

**Table 6:** Eigenvectors for the variables contributing to a principal component (PC) analysis of variable measured from four industrial hemp varieties.

Component	Variable	PC1	PC2	PC3
Proximate analysis	Moisture	−0.547	0.043	0.806
	Ash	0.456	0.670	0.393
	Crude fiber	0.555	0.066	0.148
	Protein	0.431	−0.738	0.417
Amino acids	Isoleucine	0.262	0.004	0.230
	Leucine	0.266	−0.037	0.118
	Valine	0.260	0.898	0.224
	Arginine	0.268	−0.015	−0.003
	Lysine	0.268	0.024	−0.035
	Phenylalanine	0.260	−0.125	0.174
	Threonine	0.260	−0.154	0.111
	Histidine	0.261	0.135	0.072
	Methionine	0.266	−0.077	0.076
	Alanine	0.110	0.568	0.341
	Aspartic Acid	0.124	0.462	−0.613
	Cystine	0.219	0.312	−0.368
	Glutamic Acid	0.251	−0.172	−0.267
	Glycine	0.267	0.017	0.075
	Proline	−0.153	0.506	0.333
	Serine	0.267	−0.065	−0.033
	Tyrosine	0.266	−0.054	−0.103
Fatty acids	16:0 Palmitic acid	0.269	−0.171	−0.132
	18:0 Steric acid	0.272	−0.161	0.044
	20:0 Arachidic acid	0.279	−0.127	−0.223
	22:0 Behenic acid	0.279	−0.130	−0.107
	24:0 Lignoceric acid	0.269	−0.171	−0.132
	9c 18:1 Oleic acid	0.279	−0.125	0.199
	18:2 Linoleic acid	0.117	0.399	0.373
	18:3 Alpha Linolenic acid	0.184	0.333	−0.455
	Omega 3 fatty acid	0.184	0.333	−0.455
	Omega 6 fatty acid	0.117	0.399	0.373
	Omega 9 fatty acid	0.279	−0.125	0.199
	Saturated fatty acids	0.273	−0.159	−0.088
	Monounsaturated fatty acids	0.283	−0.104	0.240
	Polyunsaturated fatty acids	0.149	0.380	−0.012
	Total 18:1 cis	0.282	−0.106	0.245
	Total cis unsaturated fatty acids	0.210	0.307	0.056
	Total fatty acids	0.273	0.159	−0.006
Minerals	Boron (B)	0.374	−0.230	0.226
	Calcium (Ca)	0.356	−0.357	−0.163
	Copper (Cu)	0.341	0.355	0.244
	Iron (Fe)	0.386	0.218	0.110
	Magnesium (Mg)	0.388	0.024	0.288
	Manganese (Mn)	0.392	0.192	−0.055
	Phosphorus (P)	−0.227	0.247	0.714
	Potassium (K)	0.172	−0.664	0.010
	Zinc (Zn)	0.287	0.327	−0.521

## Data Availability

Data sharing not applicable.
